# Naming of Stimuli in Equivalence Class Formation in Children

**DOI:** 10.1007/s40616-021-00143-8

**Published:** 2021-03-10

**Authors:** Guro Granerud, Erik Arntzen

**Affiliations:** Department of Behavioral Science, Oslo Metropolitan University, Oslo, Norway

**Keywords:** Stimulus equivalence, Naming, Individual, Common, Children

## Abstract

In the present study, two typically developing 4-year-old children, Pete and Joe, were trained six conditional discriminations and tested for the formation of three 3-member equivalence classes. Pete and Joe did not establish the AC relation within 600 trials and were given two conditions of preliminary training, including naming of stimuli with two different stimulus sets. Pete started with preliminary training with common naming of stimuli, followed by conditional-discrimination training and testing for emergent relations, and continued with preliminary training on individual naming of stimuli, followed by the same training and testing as described previously. Joe experienced the same conditions but in reversed order. Pete responded in accordance with equivalence in the second round in the condition with common naming. In the first round of testing in the condition with individual naming, he responded in accordance with equivalence. In the condition with individual naming, Joe did not respond in accordance with stimulus equivalence but established all of the directly trained relations during training. In the condition with common naming, he responded in accordance with equivalence in the first round of testing. The results from the experiment support earlier findings that both common and individual naming could facilitate the emergence of equivalence classes.

For many years, the role of naming in the formation of equivalence classes has been discussed, whether responding in accordance with stimulus equivalence depends on giving names to the stimuli or not (see Miguel & Petursdottir, 2009; Sidman, 1994). A series of experiments with children has shown that equivalence class formation and verbal behavior are connected (Barnes, McCullagh, & Keenan, 1990; Bentall, Dickins, & Fox, 1993; Devany, Hayes, & Nelson, 1986; Eikeseth & Smith, 1992; Jennings & Miguel, 2017; Ma, Miguel, & Jennings, 2016; Petursdottir, Carp, Peterson, & Lepper, 2015). In contrast, other studies have not shown such a connection between equivalence class formation and verbal behavior (Carr, Wilkinson, Blackman, & McIlvane, 2000; Lipkens, Hayes, & Hayes, 1993). However, Sidman (1994) discussed the role of naming extensively, underscoring that the naming of stimuli could facilitate equivalence class formation, but argued that equivalence class formation is not dependent on it.

When all the members of a class are given the same name, it is called common naming (Miguel, 2016). Individual or intraverbal naming occurs when all the members of a class have different names. Many studies have shown that common naming facilitates equivalence class formation (Bentall et al., 1993; Dugdale & Lowe, 1990; Eikeseth & Smith, 1992; Horne, Hughes, & Lowe, 2006; Horne, Lowe, & Harris, 2007; Horne, Lowe, & Randle, 2004; Lowe, Horne, & Hughes, 2005). In the study by Eikeseth and Smith (1992), four pre-school children with autism without other developmental disabilities were trained four conditional discriminations with arbitrary stimuli and tested for the formation of two 3-member classes. None of the children formed equivalence classes, but two of the children did so after being trained in common naming of the two stimulus sets. In the next phase, two new classes were trained with common naming, and testing assessed whether the children responded in a class-consistent manner without a prior matching-to-sample (MTS) training procedure. Two of the children responded in a class-consistent manner, and functional classes were established without the features of an equivalence class. In the third phase, named and unnamed stimuli were tested together, and one child responded in accordance with stimulus equivalence. In the last phase, a new stimulus set was trained and tested without naming the stimuli, and two children responded in accordance with stimulus equivalence.

Additionally, experiments have shown that individual naming facilitates equivalence class formation (Jennings & Miguel, 2017; Lazar, Davis-Lang, & Sanchez, 1984; Lowe & Beasty, 1987; Ma et al., 2016). Carp and Petursdottir (2015) conducted an experiment where six children, ranging in age from 5 to 7 years, were trained on six conditional discriminations (AB and AC) in a one-to-many training structure and tested for the formation of three 3-member equivalence classes. The procedure included training on individual names for each stimulus. The children were given tests for both equivalence class formation and intraverbal naming. The results showed that three children passed both tests, whereas three children did not, even when a modified equivalence test with changes made to promote intraverbal responses was conducted. Petursdottir et al. (2015) found that 5 out of 10 preschool children responded in accordance with symmetry during a visual–visual MTS test after being trained on tacting of stimuli and intraverbal responding (A1, A2, and A3 were dictated, and the children vocalized B1, B2, and B3). Four out of the five children who had already responded in accordance with symmetry during the visual–visual MTS test failed a reverse intraverbal test (B1, B2, and B3 were dictated, and the children were tested on whether they could vocalize A1, A2, and A3). Although the children had responded in accordance with symmetry during the visual–visual MTS test, four out of five were not able to respond correctly to stimuli in the reverse order of training.

In the present experiment, we wanted to explore the effects of both common and individual naming on equivalence class formation. Hence, the research question was whether two typically developing 4-year-old children would respond in accordance with equivalence with training on common and individual naming.

## Method

### Participants

Two typically developing children, Joe (3 years 9 months) and Pete (3 years 11 months), participated in the experiment. Neither child had any experience with MTS tasks beforehand. Their parents were given an informative letter, including a consent form, before the start of the study. The letter consisted of information about the project and how the training and testing would be conducted, and stated that the children could withdraw at any time. The parents were debriefed after the children finished the experiment. The parent debriefing included information about the training and testing, and they could ask questions. After each session, the children were informed that the experiment would continue the next day or over the weekend and that participating was voluntary. The children received an age-appropriate debriefing after the end of the experiment.

### Setting and Apparatus

The training was conducted 3–5 days a week in the kindergarten building, with one 15- to 20-min sessions per day. The length of the training and number of trials varied depending on how long the children wanted to continue. The sessions were kept short to ensure that the children could participate in ordinary activities together with the other children in the kindergarten. The sessions were conducted between 11:00 a.m. and 2:00 p.m., depending on other activities in the kindergarten. Two rooms with no disturbing stimuli were used. Both rooms were 3 m × 3 m with a table and a chair where the children were seated. The first room had all objects covered with a blanket. The second room had a refrigerator, a bench with a sink, a door to the toilet, a cabinet door, and an exit door, and other objects were covered with a blanket. The computer used for running the MTS experiment was placed on a table in front of the child, and the experimenter was seated behind the child. During the training on naming the stimuli, the child and experimenter were seated facing toward one another.

The computer used was a Compaq nc6320 running Windows 7 Professional 32. A custom-made program was used to run the MTS software. A Keytec touch screen (http://www.touchwindow.com/c/Magic-Touch-Keytec.html) was used to record responses.

### Design

A crossover single-subject design was conducted. The experiment included six phases. In Phase 1, the children were trained to touch the screen. In Phase 2, a presorting test was conducted. The pre-class-formation sorting test was employed to control for the partitioning of stimuli into experimenter-defined classes before training was started. In Phase 3, MTS training without explicit training on naming the stimuli was arranged. The training was discontinued if the mastery criterion was not met within 600 trials for the first trained relation (AC). The next phase employed training on naming the stimuli with two different stimulus sets. In Phase 4a, the children were trained in common naming, and in Phase 4b, the children were trained in individual naming. In Phases 5a and 5b, the children were exposed to MTS training and testing with either common or individual naming. In Phase 6, a postsorting test was conducted. The purpose of the post-class-formation sorting test was to see whether the children partitioned the stimuli in accordance with their performance on the MTS test.

**Fig. 1:**
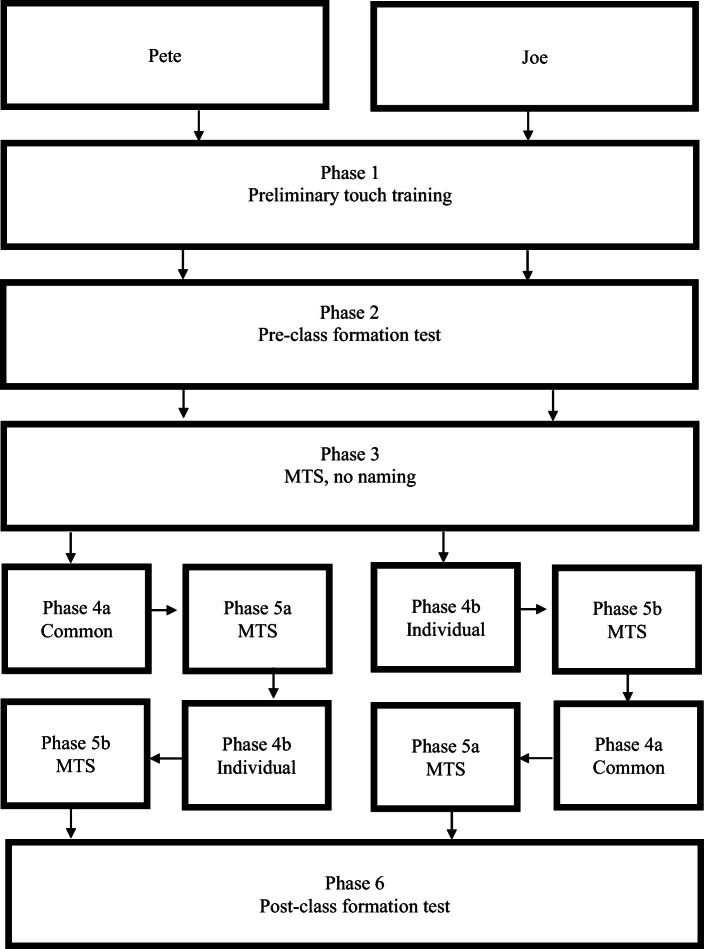
The different phases of the experiment. *Note.* MTS = matching to sample

Pete and Joe were exposed to the phases with the training on naming of stimuli and the conditional-discrimination training with common and individual naming in a different order. Pete was trained on common naming followed by individual naming, while Joe was trained on individual naming followed by common naming (see Fig. 1).

### Dependent and Independent Variables

The MTS software recorded whether the responses were correct or incorrect in accordance with the experimenter-defined classes. Touching the stimuli was measured as the dependent variable. Furthermore, correct and incorrect choices responses during MTS training and test were measured as dependent variables, as well as sorting responses in the sorting tests before and after the MTS training and test. The independent variables were the MTS with and without naming and the programmed consequences for correct and incorrect responses.

### Stimuli

**Fig. 2:**
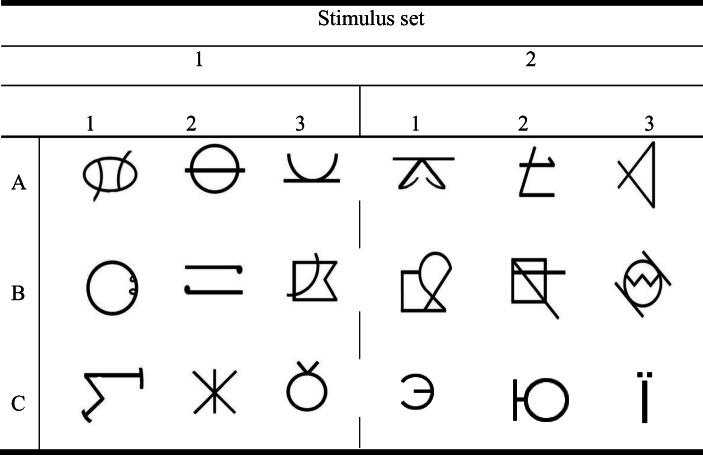
The two stimulus sets used in the present study

The abstract shapes used in this study are shown in Fig. 2. Two different stimulus sets, one for each of the two conditions, were employed.

### Training and Testing

#### Preliminary Training and Sorting

Before MTS training started, the children were trained to use the touch screen by touching numbers on the screen. The experimenter instructed the child on the numbers to touch. The preliminary training was conducted in 5-min blocks and included one break. In the pre- and post-class-formation sorting phases, the children were given the stimulus cards and instructed to “put the cards where they belong.”

#### Instruction

Before MTS training started, the following instructions were read out loud by the experimenter:

A picture will be presented in the middle of the screen. Tap it with one of your fingers. Three other pictures will appear. Tap one of them. If you tap the correct one, I will tell you by saying things like “correct” and “super.” If you tap the wrong one, I will say “wrong.” After a while, I will not tell you if it is correct or wrong. Now we can start.

If the children did not respond within 5 s to the sample or comparison, they were reminded about the task. In Phases 4a and 4b, the children were instructed at the start of each session to say the names of the stimuli out loud. During the MTS procedure, after one trial without saying the names out loud, the children were reminded to do so.

#### Training

When the sample was presented, a response to the sample stimulus was followed by the presentation of three comparisons in the corners of the screen, with one corner empty. The position of the comparisons varied from trial to trial, and all four corners were used. The sample and the comparison stimuli were presented on the screen simultaneously. The intertrial interval, the time between the response to the comparison and the next sample presentation, was 3,000 ms, and it included a 2,000-ms presentation of the programmed consequences. The length of the intertrial interval was to ensure that the children could hear the words announced as programmed consequences. The children responded to the comparison by tapping the stimulus with a finger. The programmed consequences were words such as “super,” “fantastic,” and “great” when the response was correct, said by the experimenter in an enthusiastic voice. These words had been shown to have an increasing effect on behavior in other contexts. The word “wrong” was said in a neutral voice when the response was incorrect. This word had been shown to have a decreasing effect on behavior in other contexts. Each child got a science book on the 1st day, and at the end of each session, they got a sticker to put into the science book that could be exchanged for different activities. (The Norwegian Center for Research Data did not approve that the stickers were dependent on correct responses.)

The training structure was many to one (MTO), with a serialized arrangement of baseline trials, which means that the relations were trained as AC and BC in separate blocks, followed by a mixed block of AC/BC relations (see Table 1). These blocks were conducted with 100% programmed consequences. AC and BC blocks required that all 15 trials in a block had to be correct, whereas the mixed AC/BC block required a minimum of 29 out of 30 trials (96.7%) to be correct. When the AC, BC, and mixed training was completed, the children conducted blocks with a thinning of programmed consequences of 75%, 25%, and 0% probability of programmed consequences with a 96.7% mastery criterion (see Table 1).

**Table 1:** Different blocks, trial types, programmed consequences, and minimum trials in the matching-to-sample procedure

Block	Trained/tested relations	% programmed consequences	Minimum trials
AC	A1C1 A2C2 A3C3	100	15
BC	B1C1 B2C2 B3C3	100	15
Mix	A1C1 A2C2 A3C3	100	30
Thinning	B1C1 B2C2 B3C3 A1C1 A2C2 A3C3 B1C1 B2C2 B3C3	75	30
Thinning	A1C1 A2C2 A3C3 B1C1 B2C2 B3C3	25	30
Thinning	A1C1 A2C2 A3C3 B1C1 B2C2 B3C3	0	30
Test	Baseline probes: A1C1 A2C2 A3C3 B1C1 B2C2 B3C3 Symmetry: C1A1 C2A2 C3A3 C1B1 C2B2 C3B3 Transitivity/ equivalence: A1B1 A2B2 A3B3 B1A1 B2A2 B3C3	0	90

#### Naming

Joe and Pete were trained in naming the stimuli before a new round of conditional-discrimination training (Phases 4a and 4b). They were exposed to the two naming conditions but in reverse order. The names used in the naming conditions were nonsense syllables: consonant-vowel-consonant combinations, as shown in Table 2. The names were trained by the experimenter, who showed a card with a stimulus to the participant and said the name. The child had to echo the name in the presence of the stimulus card. If the response was correct, the consequence was “correct,” and if the response was incorrect, the consequence was “incorrect.” Naming of the stimuli was arranged with the presentation of stimuli in different groups, followed by a mix of stimuli at the end. Training of names of all the A stimuli trained was trained first, followed by training of names of all the C stimuli, and finally training of names of all the B stimuli. Then, we employed a mixed training of names of all A stimuli and one C stimulus, a mixed training of names of the rest of the C stimuli and all B stimuli, and finally a mixed training of names of all the A, B, and C stimuli, respectively. To reduce the probability that classes would be established before MTS training and testing, we did not train the stimuli in separate classes (A1B1C1, A2B2C2, and A3B3C3). The training was conducted in sessions of 5–10 min each, 3–5 days a week. All stimuli in one training set were presented five times in a session before a break. After three sessions with 100% correct naming of the stimuli in one group of stimuli, the experimenter showed only the card with the stimulus and prompted the name if necessary. The mastery criterion was three consecutive sessions with 100% correct responses without prompts. After the names were established for all stimuli trained, the child started a new round of conditional-discrimination training and testing in Phase 5.

**Table 2:** Stimuli names used in training of common and individual naming

Stimulus set	
1	2
**FAB**	**KIL**
**SEM**	**LIS**
**MIP**	**JOP**
NAP	PAV
BAK	VIM
PAG	MIR
SIB	SAP
VUR	PON
LOR	NOS

*Note*. The names in bold were used in the common naming condition

#### Testing

The testing phase included 90 trials, with 30 trials of baseline, symmetry, and transitivity/equivalence, respectively. The criterion for responding in accordance with equivalence was 96.7% correct on each of these probes. If the child did not meet the criterion, the AC/BC mixed block was retrained before a second testing phase was introduced.

#### Breaks During Training and Testing

In both training and testing, a break was programmed after every fifth trial, and the children could then choose if they wanted the break or not. During breaks, the children could choose from preferred activities and different games that were available or games on a cell phone, such as car racing, drawing, or completing a puzzle. The experimenter stayed with the children during the breaks, helping out with the activities. The breaks took place in the same room as the experiment.

#### Interobserver Agreement

Interobserver agreement (IOA) was determined for the pre- and post-class-formation sorting phases, with 100% agreement. IOA was calculated as the number of agreements divided by the number of agreements plus the number of disagreements, multiplied by 100. In addition, IOA was calculated for 10% of the training sessions with naming the stimuli (Phases 4a and 4b) with an agreement of 100%. The experimenter and one observer performed IOA.

## Results

The data from the pre-pretraining phase (Phase 1) are not presented. None of the children sorted the stimuli according to the experimenter-defined classes in Phase 2.

### Training Phase 3

Neither Pete nor Joe established the AC relation within 600 trials.

### Naming of Stimuli (Phases 4a and 4b)

Pete needed 13 sessions for the common naming procedure and 19 sessions for the individual naming procedure, whereas Joe needed 28 sessions for the individual naming procedure and 11 sessions for the common naming procedure.

### Phase 5a to Phase 5b

#### Responding During Training and Testing

**Fig. 3:**
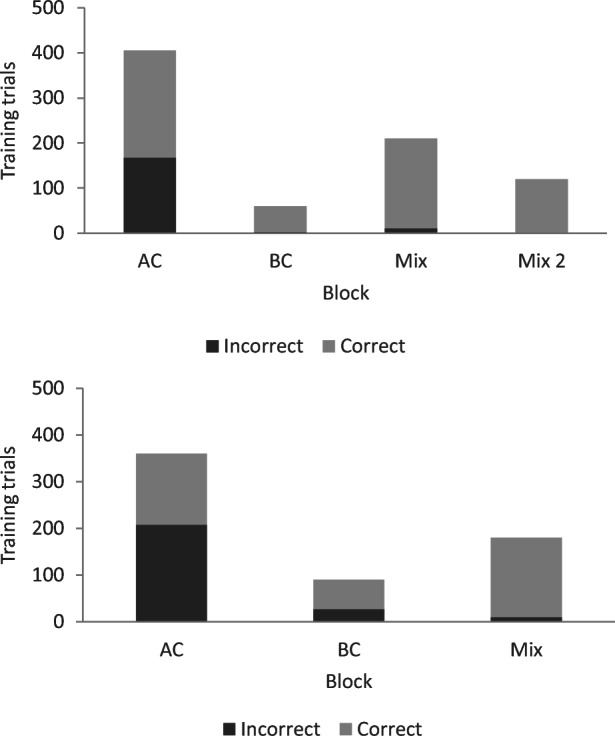
Number of correct and incorrect trials per block during training in phases 5a and 5b for Pete. *Note.* The numbers for Phase 5a with common naming are presented in the upper panel, and the numbers for Phase 5b with individual naming are presented in the lower panel

In the common naming condition Pete met the mastery criterion for the AC relation after 405 trials and established the BC relation after 60 training trials and the mix of AC and BC relations with thinning of programmed consequences after 210 trials. He met the criterion for training after 675 training trials (see the three left bars in the upper panel in Fig. 3).

**Fig. 4:**
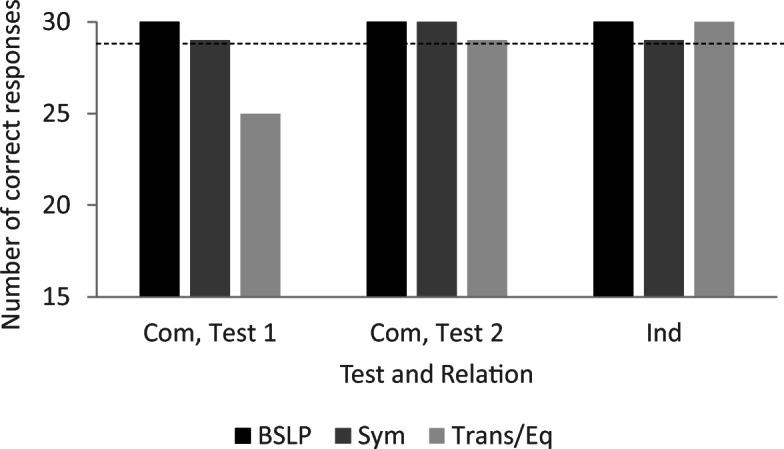
Number of correct responses for each relation during testing for Pete. *Note.* All relations included 30 test trials, and the criterion for responding in accordance with equivalence was 29/30 for each relation. BSLP = baseline probes; Sym = symmetry; Trans/Eq = transitivity/equivalence; Com = common; Ind = individual

In Test 1, Pete did not respond correctly to the transitivity/equivalence probes; however, baseline performance was intact, and he responded in accordance with symmetry (see Fig. 4). He was then exposed to retraining and met the mastery criterion after 120 training trials with no errors (see the right-most bar in the upper panel in Fig. 3). In Test 2, he responded in accordance with stimulus equivalence (see Fig. 4).

In the condition with individual naming, Pete established the AC relation within 360 trials, the BC relation within 90 trials, and the mix of AC and BC relations within 180 trials (see the lower panel in Fig. 3). He met the criterion for training after 630 training trials. In the test for emergent relations, Pete responded in accordance with stimulus equivalence (see Fig. 4).

#### Patterns of Responding

**Fig. 5:**
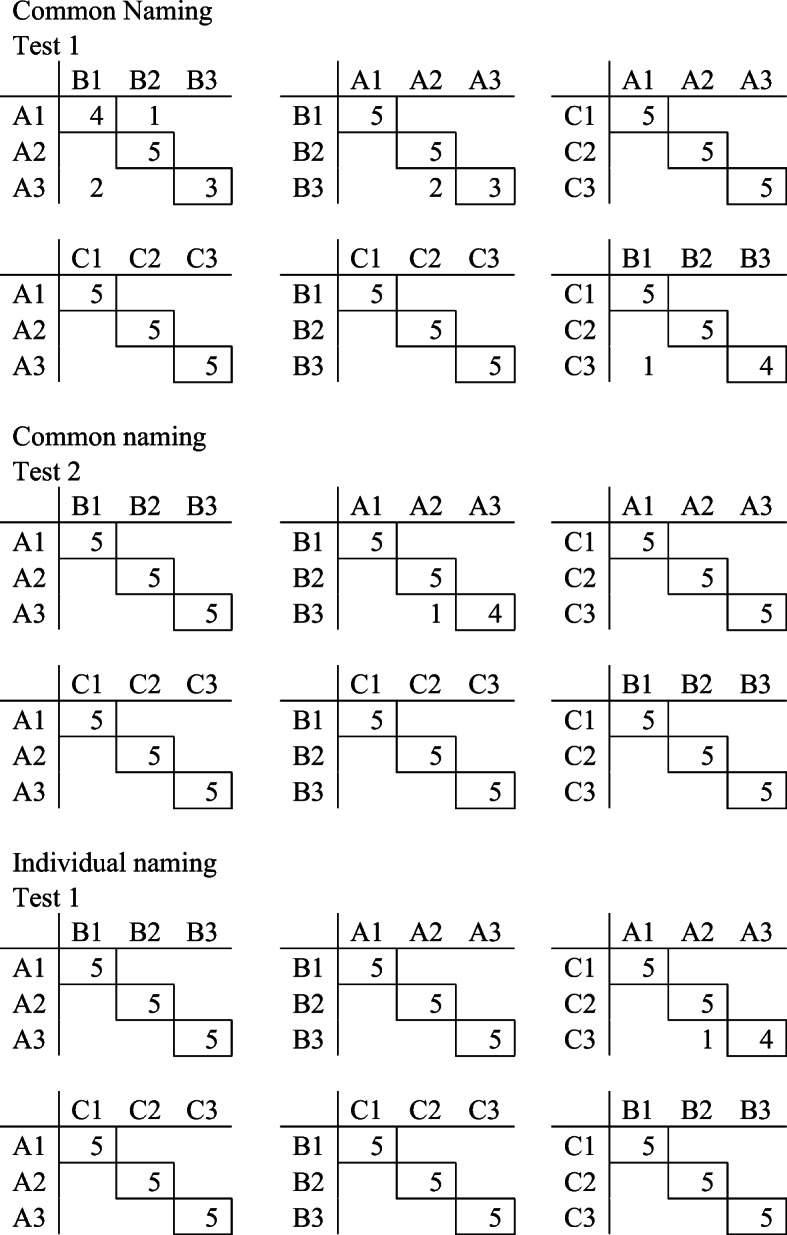
Distribution of responses during testing for Pete. *Note.* The horizontal letter/number combinations are the samples presented on the screen. The vertical letter/number combinations are the chosen comparisons. The framed numbers are the number of responses correct in accordance with experimenter-defined classes. The numbers without a frame are incorrect responses

**Fig. 6:**
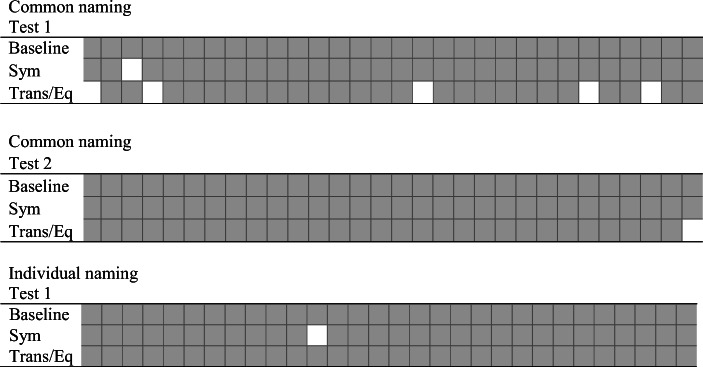
Distribution of correct and incorrect responses during testing over time for Pete. *Note.* Gray squares represent correct responses. White squares represent incorrect responses. *E*ach tested relation consisted of 30 trials, as presented in the figure. Sym = symmetry; Trans/Eq = transitivity/equivalence

Class-consistent responding and the distribution of correct and incorrect responses were analyzed in the test. As shown in Fig. 5, there was no systematic pattern of errors in the testing phase. Figure 6 shows the distribution of responses during testing. Incorrect responses during Test 1 in the common condition were not clustered, and for Test 2, there were no errors. Furthermore, for the test in the individual condition, there was only one incorrect response.

### Phase 5b to Phase 5a

#### Responding During Training and Testing

**Fig. 7.**
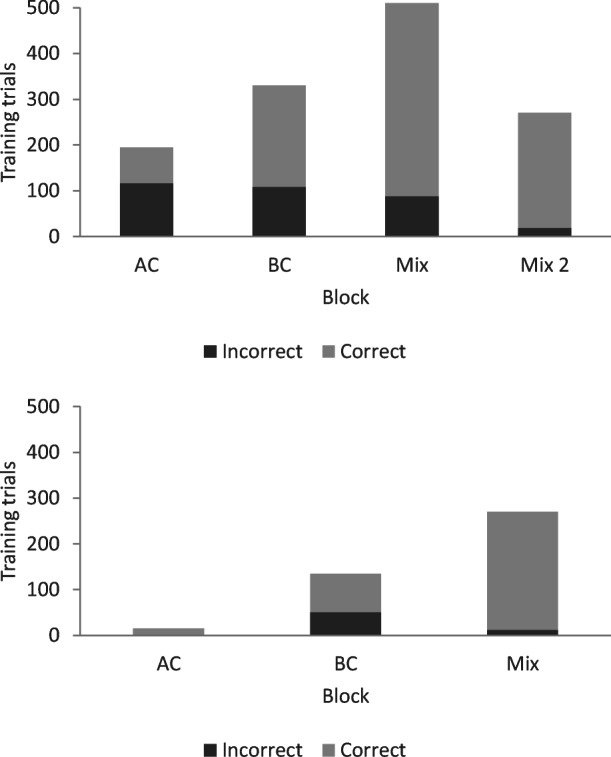
Number of correct and incorrect trials per block during training in phases 5b and 5a for Joe. *Note.* Phase 5b with individual naming is presented in the upper panel, and Phase 5a with common naming is presented in the lower panel. Due to a programming error, Joe received 30 extra training trials when establishing the AC relation. These training trials are not included in the figure

In the condition with individual naming, Joe established the AC relation after 195 training trials, the BC relation after 330 trials, and the mix of AC and BC relations with thinning of programmed consequences after 510 trials (see the three left bars in the upper panel in Fig. 7), thus meeting the criterion for training after 1,035 trials.

**Fig. 8:**
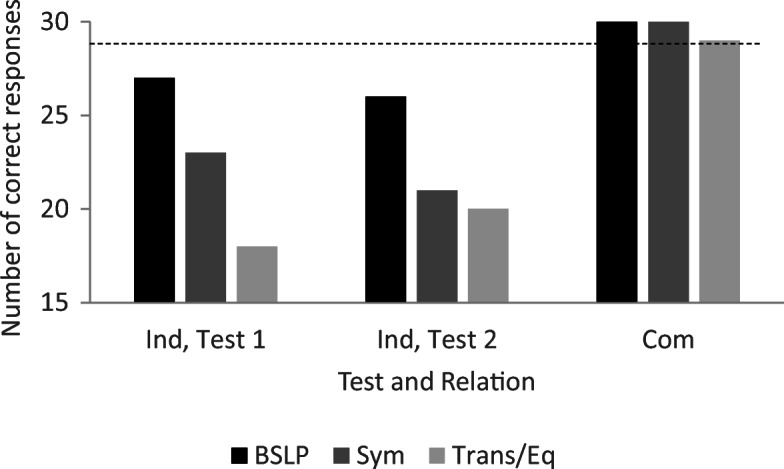
Number of correct responses for each relation during testing for Joe. Note. All relations included 30 test trials, and the criterion for responding in accordance with equivalence was 29/30 for each relation. BSLP = baseline probes; Sym = symmetry; Trans/Eq = transitivity/equivalence; Ind = individual; Com = common

In the test for emergent relations, Joe did not respond correctly on baseline trials and did not respond in accordance with symmetry or transitivity/equivalence (see Fig. 8). He was then exposed to retraining and a second test. He met the mastery criterion for the mix of AC and BC relations in training after 270 trials (see the right-most bar in the upper panel in Fig. 7). However, he did not respond in accordance with the experimenter-defined classes in testing (see Fig. 8).

In the conditional-discrimination training with common naming, Joe established the AC relation after 15 training trials, the BC relation after 135 training trials, and the mix of AC and BC relations with thinning of programmed consequences after 270 trials. He met the criterion for training after 420 trials (see Fig. 7). During testing, baseline performance was intact, and Joe responded in accordance with stimulus equivalence (see Fig. 8).

#### Patterns of Responding

**Fig. 9:**
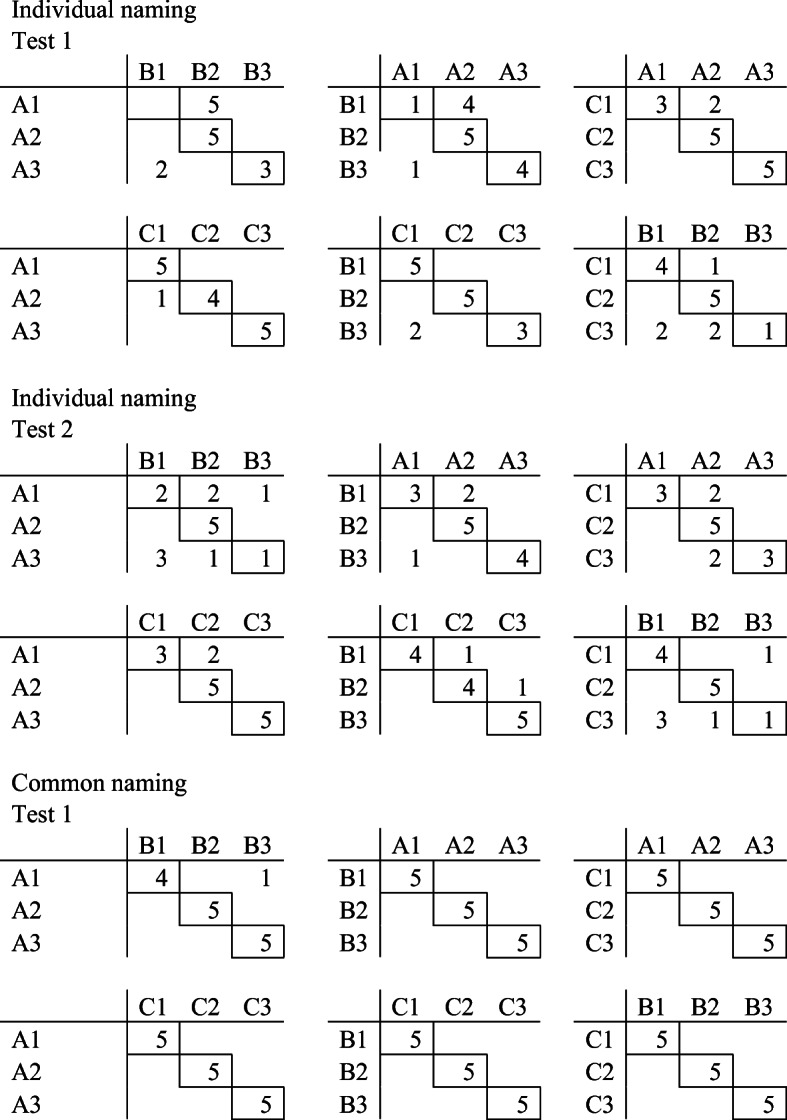
Distribution of responses during testing for Joe. *Note.* The horizontal letter/number combinations are the samples presented on the screen. The vertical letter/number combinations are the chosen comparison. The framed numbers are the number of responses correct in accordance with experimenter-defined classes. The numbers without a frame are incorrect responses

Most of the errors during the testing for equivalence with the individual naming procedure were unsystematically distributed except when A1 and B1 were presented as samples during Test 1 (see Fig. 9). Hence, when A1 was presented as the sample, Joe responded to B2 in all five trials, and when B1 was presented as the sample, he responded to A2 in four of five trials. In Test 2, responding to comparisons was more random. When A1 was presented as the sample, B1 was selected two times, B2 was selected two times, and B3 was selected one time. When B1 was presented as the sample, A1 was selected three times and A2 was selected two times. Baseline probes that were intact in the first test were no longer stable. For example, during Test 1, Joe responded to comparison C1 five out of five times when A1 was presented as a sample, whereas during Test 2, he responded to C1 three times and C2 two times. During the test in the common naming condition, Joe responded incorrectly only once.

**Fig. 10:**
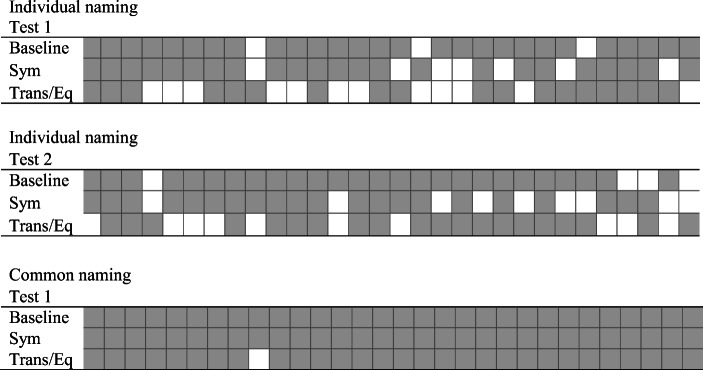
Distribution of correct and incorrect responses during testing over time for Joe. *Note.* Gray squares represent correct responses. White squares represent incorrect responses. Each test relation consisted of 30 trials, as presented in the figure. Sym = symmetry; Trans/Eq = transitivity/equivalence

Figure 10 shows how the correct and incorrect responses were distributed in the individual and common naming conditions. Joe showed an unsystematic distribution of correct and incorrect responses over time.

### Sorting (Phase 6)

Pete sorted the stimuli correctly in the post-class-formation sorting test. Joe sorted the stimulus sets correctly in the post-class-formation sorting tests. Joe sorted the stimuli correctly for Set 1 with individual naming, even if he did not respond in accordance with equivalence in any of the MTS tests with individual naming.

## Discussion

### Number of Training Trials

The number of training trials decreased from the first to the second condition. This effect was most pronounced for Joe, starting with the individual naming condition. It is important to emphasize that the individual naming procedure was more time consuming because nine individual names were trained, compared to the common naming procedure, where only three names were trained.

### Equivalence Class Formation

The results show that two 4-year-old children did not meet the experimenter-defined criterion in training of the conditional discriminations. However, they established the conditional discriminations after been trained in naming the stimuli. Hence, both Pete and Joe responded in accordance with stimulus equivalence in one or two of the conditions with common or individual naming. The results from the present experiment give some support to previous research that found that naming of stimuli can facilitate equivalence class formation (Bentall et al., 1993; Carp & Petursdottir, 2015; Eikeseth & Smith, 1992; Jennings & Miguel, 2017; Ma et al., 2016; Petursdottir et al., 2015).

### Possible Effect of Order

There were differences in responding in accordance with equivalence depending on the naming conditions—common or individual naming—the children were exposed to. Pete, who was exposed to common naming first, did respond in accordance with equivalence in Test 2 and had less than half of the training trials Joe had in the individual naming condition. Pete also responded in accordance with equivalence in Test 1 in the individual condition. It could be that exposure to the common naming condition first made the individual naming condition easier. Joe, who was exposed to individual naming first, did not have intact baseline performance during testing and did not respond in accordance with symmetry and transitivity/equivalence in this condition. However, when Joe was exposed to the common naming of stimuli, he responded in accordance with stimulus equivalence in the first test. The number of training trials was also reduced by 50% in this condition compared to the individual condition; that is, establishing the necessary conditional discriminations demanded fewer training trials.

### Naming as a Facilitator for Equivalence Class Formation

Earlier studies support the findings in this article showing that naming could facilitate equivalence class formation. Given the possible effect of order as discussed previously, one interpretation of the data is that common naming facilitated equivalence class formation slightly better than individual naming. In common naming, two similar names are linked together (e.g., FAB-FAB), whereas in individual naming, different names are linked together (e.g., FAB-SEM). Individual naming can facilitate equivalence class formation when the children produce sequences of names (e.g., FAB-SEM-MIP) that are established, and then the child repeats this chain of names from the presentation of a sample to the choice of a comparison, establishing an intraverbal relation between tacted stimuli (Lowe & Beasty, 1987; Ma et al., 2016; Miguel, 2016).

Training a common response to three or more stimuli in a class can establish a stimulus-response-stimulus chain. Saunders (1989) argued that a stimulus-response-stimulus chain is established when using common names, and that the defining characteristics of an equivalence class have not been met. A stimulus-response-stimulus chain can be established when responding to a comparison is controlled by the same topography (here, the same name) as that which is evoked by the sample. In the present study, all of the members in one class were named “SEM.” Therefore, if a stimulus-response-stimulus chain had been established, the comparison would control the response “SEM.” When the three comparisons are presented, the response “SEM” controls the choice of the comparison with the same name, even if the relation is not directly trained. It is essential to emphasize that both Joe and Pete produced incorrect responses during conditional-discrimination training in the common condition, which could indicate that chains were not established just because of the naming training. Pete did not respond in accordance with equivalence before the second test in the common naming condition. We argue that these results suggest that the relations were not established beforehand. Furthermore, when the stimulus names were trained, they were not trained as an experimenter-defined class. The A stimuli were trained separately, as were B and C stimuli, and then mixed. However, if the stimuli had been taught in classes—that is, A1, B1, and C1; A2, B2, and C2; and A3, B3, and C3—then one could argue that the classes were established beforehand.

### Sorting of Stimuli

A stimulus-sorting test is a quick way to test for class partitioning. Additionally, stimuli sorting after the MTS test for emergent relations has been shown to correlate with the results from the test. In some cases, the delayed emergence of equivalence classes is also revealed (Arntzen et al., 2017; Arntzen, Norbom, & Fields, 2015; Fields, Arntzen, & Moksness, 2014). In the present study, neither child sorted the stimuli into the correct classes before training and testing. In the condition with individual naming, Joe did not respond in accordance with stimulus equivalence during the test, but he sorted the cards after the MTS test. This finding may be an example of delayed emergence, but there are some limitations in interpreting this as an example of delayed emergence. A sorting test with stimulus cards differs from an equivalence test in an MTS format because the participant has all the stimuli available at the same time and can scan back and forth among the stimuli to figure out which ones belong together. However, the distribution of correct and incorrect responses over time indicated that there was not a delayed emergence during the MTS test. For example, in a study by Spradlin, Cotter, and Baxley (1973), delayed emergence relations appeared after repeated testing, and when a participant does not respond in accordance with equivalence after naming the stimuli, repeated testing should be conducted to control for this.

Joe emitted several systematic errors during Test 1 in the individual naming condition, which implies that classes other than those defined by the experimenter were established (Arntzen, Nartey, & Fields, 2015; Eilifsen & Arntzen, 2009; Mensah & Arntzen, 2017). During Test 2, these errors were not systematic. Although he did not respond in accordance with equivalence, the participant-defined classes were no longer consistent.

### Limitations and Further Research

There are several limitations to the present study. First, the sessions in the present experiment were conducted without controlling for session length or the number of trials per day/session. The arrangement in the present experiment was done because the children should decide how long they wanted to participate per session. However, it is important to emphasize that the length of the sessions varied, but this variation did not seem to influence the results.

Second, we did not check the effect of the programmed consequences for the touching responses in the MTS tasks directly. However, the words used as the programmed consequences in this experiment had shown increasing or decreasing effects on behavior in other contexts. The words were spoken out loud by the experimenter, with an enthusiastic (for correct responses) or neutral (for incorrect responses) voice. There is a possibility that the programmed consequences alone had a limited reinforcing effect on establishing the baseline relations. If a token system with items like preferred toys and games had been used contingent on correct responses, the baseline relations probably could have been established faster. Such an arrangement was not employed due to restrictions from the Norwegian Center for Research Data that required that the children receive the same amount of benefits.

Third, the number of trials was quite large in the conditional-discrimination training. Additionally, research with adults using an MTO training structure with abstract shapes has sometimes demonstrated a high number of training trials (Arntzen & Hansen, 2011). Other experiments with older children employing a preliminary training phase have the present study that the number of trials to mastery was lower than in the previous study (e.g., Pilgrim, Jackson, & Galizio, 2000; Smeets & Barnes-Holmes, 2005). Researchers should consider conducting preliminary training to familiarize the children with the task, reducing the large number of errors at the start of the MTS training. Such preliminary training could be identity matching with colors.

Both the short session length and the frequent breaks (after every fifth trial) could have hindered the flow in the establishment of the conditional discriminations. Furthermore, these variables could be responsible for the large number of trials in the present study. It is challenging to arrange procedures as in the present study with 4-year-old children without having short sessions and frequent breaks. Additionally, we had to consider that the children should not be excluded from other activities in the kindergarten.

Fourth, the effect of the naming conditions could be confounded. To control for the effect of the MTS procedure alone, extended sessions with extended training and testing could be used to study the effect of repeated exposure to the procedure. An effect of order should be observed as a reduction of the number of trials to meet the criterion in the second condition. Such a reduction in the number of training trials was observed especially for Joe.

Finally, only two children served as participants in the experiment, and replications should be conducted to control for the differences demonstrated in the present experiment.

Another important line of research is the effect on stimulus class formation of having children make up names themselves, rather than using experimenter-defined names as in the present study.

### Summary

Neither Pete nor Joe established the necessary conditional discriminations without any training on naming the stimuli. Pete, who started with common naming, responded in accordance with equivalence in the second test in the first condition and the first test in the individual naming condition. Joe, who started with the individual naming condition, did not respond in accordance with equivalence but did so after the common naming condition.
